# Pediatric trauma mortality by type of designated hospital in a mature inclusive trauma system

**DOI:** 10.4103/0974-2700.76824

**Published:** 2011

**Authors:** Rachid Amini, André Lavoie, Lynne Moore, Marie-Josée Sirois, Marcel Émond

**Affiliations:** 1 Unit of Traumatology, Emergency Medicine and Intensive Care, Research Centre of CHA (Enfant-Jésus Hospital), Quebec (QC), Canada; 2 Department of Preventive and Social Medicine, Laval University, Quebec (QC), Canada; 3 Department of Family Medicine and Emergency Medicine, Laval University, Quebec (QC), Canada

**Keywords:** Adult trauma center, logistic regression, pediatric trauma center, trauma mortality

## Abstract

**Background:**

Previous studies have shown divergent results regarding the survival of injured children treated at pediatric trauma centers (PTC) and adult trauma centers (ATC).

**Aims:**

(1) To document, in a regionalized inclusive trauma system, at which level of trauma centers were the injured children treated and (2) to compare the in-hospital mortality over five levels of trauma care, ranging from pediatric level I trauma centers (PTC) to designated local trauma hospitals (level IV) for the whole study sample and for subgroups of severely injured children and head trauma.

**Materials and Methods:**

A retrospective analysis included data on 11,053 injured children (age ≤16 years) treated between April 1998 and March 2005 in 58 designated trauma hospitals in the province of Quebec, Canada. Multiple imputation was used to handle missing physiological data and multivariate logistic regression was used to compare mortality over levels of care.

**Results:**

PTC treated 52.2% of the children. Children treated at PTC were more often transferred from another hospital (73%) and were more severely injured. ATC level I, II, III and IV centers treated, respectively, 3.0%, 16.2%, 24.3% and 4.3% of children. Compared with children treated at a PTC, the risk of mortality was higher for children treated at each other ATC, i.e. level I (adjusted odds ratio [OR] = 3.1; 95% confidence interval [CI]: 1.3–7.5), level II (OR = 2.5; 95% CI: 1.3–5.0), level III (OR = 5.2; 95% CI: 2.1–13.1) and level IV (OR = 9.9; 95% CI: 2.4–41.3). Similar findings were observed among the subsamples of children who were more severely injured (Injury Severity Score >15) and who sustained head injuries.

**Conclusions:**

In our trauma system, PTC cared for more than half of the injured children and patients treated there have better survival than those treated at all other levels of ATC.

## INTRODUCTION

Injury remains the leading cause of child death in developed countries.[1] In 2003, injuries represented, among US children, 55%, 53% and 60% of all-cause mortality in the age groups 1–4 years, 5–9 years and 10–14 years, respectively.[2] In the province of Quebec (Canada), pediatric injuries are also the most common cause of death, although their rate is lower than that in the US. In 2003, injuries accounted for 45% of the mortality among Canadian children aged 5–14 years and for 32% of the mortality among children aged 1–4 years.[3]

Pediatric trauma centers (PTC) were established to provide optimal care to injured children. However, given the relative scarcity of PTC, much of the care provided to pediatric trauma patients is performed in adult trauma centers (ATC). PTC are usually located in major urban areas while many events occur in more rural settings where distance often hinders direct transport to the PTC.[4,5] Most studies evaluating pediatric trauma care have compared the outcome of a single ATC or PTC to a large, multicenter reference population using the TRISS methodology.[6] While some investigators have reported improved survival for injured children treated at a PTC compared with the national norms,[7–9] other authors have demonstrated comparable outcomes for children treated at an ATC to national standards.[10–14]

Only a few authorshave investigated the impact of trauma center type on pediatric mortality by comparing several ATC to several PTC.[15–18] These studies have reached contradictory conclusions. Nakayama *et al*.[15] reported that injured children had improved survival when treated at PTC and urban ATC compared with rural ATC. Potoka *et al*.[16] found improved survival for children treated at PTC and ATC with added qualifications in pediatrics than those treated at level I or level II ATC. On the other hand, Sherman *et al*.[17] reported improved survival for children treated at level I ATC and ATC with added qualifications in pediatrics when compared with those treated at PTC or level II ATC. Conversely, Osler *et al*.[18] found no significant difference in pediatric trauma mortality between ATC and PTC. In addition to the controversy regarding where injured children have the highest survival, a debate has focused on the benefit of “inclusive” trauma systems as opposed to “exclusive” systems.[19,20] The concept of “inclusive trauma systems” was based on the participation in a given territory of all acute care hospitals to care for all injured patients. Acute care facilities in an inclusive system are categorized to different levels according to their trauma care ability. Throughout the system, severely injured patients may be first seen in a local hospital and then transferred to a higher level of care. This system concept differs from the “exclusive trauma system” in which trauma care is organized around a relatively few high-level trauma centers that deliver definitive care to major trauma victims.

The objective of this study was to determine at which level of care are treated pediatric patients aged 16 years or less in a regionalized inclusive trauma system serving an entire Canadian province and to compare the pediatric in-hospital mortality according to the five levels of care (PTC, level I, level II, level III and level IV ATC) for the entire study group and for subsamples of severely injured children and those with head injury.

## MATERIALS AND METHODS

### Study design

We performed a retrospective study based on the Quebec Trauma Registry (QTR) to examine the effect of trauma center type on pediatric mortality. Ethics approval was obtained from the institutional research ethics committee.

### Study setting and population

The province of Quebec, Canada, has a population of 7.5 million spread over 1.7 million km^2^ (656,370 miles^2^). A regionalized inclusive trauma system was established in 1992 and includes four levels of designated trauma centers according to criteria similar to those of the American College of Surgeons (see appendix). [21] The Quebec trauma system includes two PTC, four level I, four level II, 20 level III and 28 level IV ATC. Each hospital is officially designated by the Ministry of Health according to recommendations following a periodic external accreditation review and all have signed standing agreements with a level I trauma center for inter-hospital transfers.

Both PTC are situated in Montreal (Ste-Justine Hospital and the Montreal Children’s Hospital). In addition, the level I ATC in Quebec City (Enfant-Jesus Hospital) has added qualifications in pediatric trauma care. As PTC and ATC with commitment in pediatrics are considered to offer similar quality of care,[22,23] this particular ATC was considered as a PTC for this study.

According to prehospital protocols specific to the Montreal area (population of 2.2 million), all pediatric (i.e., ≤16 years of age) trauma patients are taken directly to one of the two PTC thus avoiding the level I and level III ATC of the area. Elsewhere in the province, all designated centers are at a distance ranging from 33 to 1,059 km (i.e., 20.5–658 miles) from the closest level I trauma center. These distances imply that a number of severely injured children are first taken to an ATC or a nondesignated hospital and later transferred if considered appropriate.

The study population was drawn from the QTR, a standardized database centralized at the Ministry of Health, where the data are subject to automatic validity assessment procedures. Data entry is performed by qualified medical archivists at each hospital during the admission and is completed after patient discharge. Although the QTR data have not been validated by audit, several measures are taken to ensure high data quality and homogeneity between centers. First, all medical archivists follow a standard training program and meet annually for additional training. Second, an electronic forum of coding queries is under continuous circulation among archivists and third, meetings are held three-times a year among registry representatives with specific questions. The QTR is mandatory for all 58 designated hospitals. Trauma patients are included in the QTR if they meet any of the following criteria: hospital stay greater than 2 days, admission to the Intensive Care Unit, transfer from another hospital or death.

All patients aged 16 years or younger from April 1, 1998 to March 31, 2005 were identified. This age cut-off was selected as it is the threshold used for PTC transfer in the Montreal prehospital system. Deaths on arrival at the emergency room (*n* = 147) were excluded as information concerning their injury severity is too limited and there was no opportunity for appropriate surgical care or transfer. A total of 11,053 observations were available for data analysis. These were stratified according to the level of the trauma center in which they were treated: PTC, adult level I, II, III or IV. Patients transferred from one hospital to another were classified in the last treating hospital.

### Outcome measures

In-hospital mortality was the outcome measure obtained from the QTR.

### Data analysis

To compare mortality over levels of care, we used logistic regression to generate odds ratios (OR) and their 95% confidence intervals (CI). These were adjusted for age, initial Emergency Department (ED) Glasgow Coma Scale (GCS) score,[24] initial ED systolic blood pressure (SBP), initial ED respiratory rate (RR) and the New Injury Severity Score (NISS).[25] As penetrating trauma represents only 1.3% of the QTR patient population, we did not adjust for the type of trauma (blunt/penetrating) in our model. Level of care was introduced into the model using four dummy variables with PTC as the reference category. Age was included as a categorical variable: <1 year, 1–4 years, 5–9 years and 10–16 years (reference category). These age categories were selected based on common mechanisms of injury and physiological vital signs. SBP and RR were entered according to the categories used in the Revised Trauma Score (RTS).[26] The NISS and the GCS score were included as continuous variables as they were linear in the logit of mortality. RR and SBP were included as dummy variables on five categories as used in the RTS. More complex functions such as fractional polynomials or cubic splines were not used as the results were similar and as they complicate the presentation of results. On the other hand, five categories on quantitative variables have been found to be sufficient for control of confounding.[27] Clustering of patients within centers was verified using a random intercept logistic model, but was found not to be a problem. The covariance parameter estimate (standard error) for hospital was 0.4195 (0.3628), indicating that there was no significant cluster effect. The outcome model is specified in the appendix.

Multiple imputation[28] was applied to handle missing physiological data (see appendix). Predictive accuracy of the model was evaluated with a measure of discrimination, i.e. the area under the receiver operating characteristics (ROC) curve, which shows the ability of the model to correctly classify fatalities (or survivors). The area under the ROC curve value varies between 0 and 1, where 0.5 indicates that discrimination is equivalent to chance alone and 1.0 indicates perfect discrimination. Statistical significance was set at P <0.05. All analyses were performed with SAS statistical software (Cary, version 9.1).

Some trauma deaths are inevitable and, for such trauma patients, interhospital transfers may be impossible. In order to rule out the effect of inevitable deaths, a secondary analysis was performed where early deaths (i.e., those occurring within the first 6 h of admission) were excluded.

Finally, to rule out the possibility of contamination with minor trauma, we also performed two other sensitivity analyses where the subsamples were restricted to children who were severely injured (Injury Severity Score (ISS)[29] =15) and those with head injury (the most common cause of death among injured children). Severely injured children were stratified by ISS instead of NISS since ISS strata have been defined and are in common use in trauma research. Children with head trauma were identified with Abbreviated Injury Scale codes.[30]

## RESULTS

The characteristics of patients by trauma center type are presented in Table 1. PTC cared for 52.2% of children, while the remainder were treated at one of the ATC. Children treated at PTC were significantly younger and more often transferred than those treated at ATC. On the other hand, patients treated at level I ATC were more frequently teenagers and more severely injured compared with those treated at other levels of care. Injured children treated at adult level I and level IV centers had the highest crude mortality while those treated at PTC had the lowest crude mortality.

**Table 1:** Characteristics of pediatric trauma patients by trauma center type

	PTC *n* (%)	Level I *n* (%)	Level II *n* (%)	Level III *n* (%)	Level IV *n* (%)	*P*-value*
No of patients (*n* = 11,053)	5,770 (52.2)	331 (3.0)	1,790 (16.2)	2,690 (24.3)	472 (4.3)	-
Age						
<1 year	454 (7.9)	9 (2.7)	94 (5.3)	98 (3.6)	14 (3.0)	*P* < 0.0001
1–4 years	906 (15.7)	19 (5.7)	231 (12.9)	277 (10.3)	47 (10.0)	
5–9 years	1,718 (29.8)	41 (12.4)	456 (25.5)	602 (22.4)	104 (22.0)	
10–16 years	2,692 (46.7)	262 (79.2)	1,009 (56.4)	1,713 (63.7)	307 (65.0)	
Male/female ratio	2.0	2.6	2.2	2.5	1.8	-
Transfers from other hospitals	4,220 (73.1)	91 (27.5)	1,019 (56.9)	1,042 (38.7)	77 (16.3)	*P* < 0.0001
ISS						
1–8	2,428 (42.1)	103 (31.3)	778 (43.5)	1,395 (51.9)	267 (56.6)	*P* < 0.0001
9–15	2,307 (40.0)	141 (42.6)	727 (40.6)	1,065 (39.6)	142 (30.1)	
16–24	524 (9.1)	37 (11.2)	167 (9.3)	127 (4.7)	39 (8.3)	
25–49	493 (8.5)	36 (10.9)	113 (6.3)	90 (3.4)	22 (4.7)	
50–75	18 (0.4)	14 (4.2)	5 (0.3)	13 (0.5)	2 (0.4)	
GCS†						
13–15	4,603 (79.8)	241 (72.8)	1,452 (81.1)	2,346 (87.2)	397 (84.0)	*P* < 0.0001
9–12	727 (12.6)	45 (13.6)	233 (13.0)	235 (8.7)	42 (9.0)	
3–8	440 (7.6)	45 (13.6)	105 (5.9)	109 (4.1)	33 (7.0)	
Injuries by body region						
Head, neck and face	2,575 (44.6)	155 (46.8)	691 (38.6)	617 (22.9)	175 (37.1)	*P* < 0.0001
Thorax	354 (6.1)	60 (18.1)	127 (7.1)	127 (7.1)	46 (9.8)	
Abdomen and pelvis	653 (11.3)	59 (17.8)	234 (13.1)	433 (16.1)	129 (27.3)	
Extremities	3,574 (61.9)	231 (69.8)	1,134 (63.4)	2,039 (75.8)	252 (53.4)	
Deaths						
Direct admission	22 (1.4)	16 (6.7)	27 (3.5)	79 (4.8)	28 (7.1)	*P* < 0.0001
Transfers	78 (1.8)	7 (7.7)	14 (1.4)	4 (0.4)	0 (0)	
Total	100 (1.7)	23 (7.0)	41 (2.3)	83 (3.1)	28 (5.9)	

ISS, INJURY SEVERITY SCORE;

* X^2^ TEST ANALYSIS

† AFTER MULTIPLE IMPUTATION

**Figure 1:**
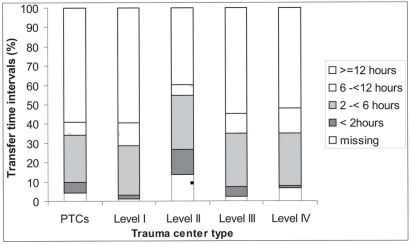
Transfer times to definitive hospitals according to trauma center type

Overall, 14.2% of the injured children were initially taken to PTC [Table 2]. Adult level I, II and III centers retained, respectively, 37%, 98% and 52% of their pediatric trauma patients. When they transferred patients, they were more likely to do so to PTC. Level IV centers, on the other hand, transferred their patients to either PTC or level III centers. Transfer delays are presented in Figure 1. With the exception of level II centers, all levels of care received nearly 50% of the transferred patients more than 12 h after admission at the referring hospital.

**Table 2:** Distribution of injured children according to the initial and final hospital

Final hospital						
	PTC *n* (%)	Adult level I *n* (%)	Level II *n* (%)	Level III *n* (%)	Level IV *n* (%)	Total *n* (%)
Initial hospital						
PTC	1,564 (14.2)	2 (0.02)	3 (0.03)	2 (0.02)	0	1,571 (14.2)
Adult level I	409 (3.7)	241 (2.2)	1 (0.01)	1 (0.01)	0	652 (5.9)
Level II	16 (0.1)	2 (0.02)	773 (7.0)	1 (0.01)	0	792 (7.2)
Level III	1,491 (13.5)	34 (0.3)	98 (0.9)	1,728 (15.6)	3 (0.03)	3,354 (30.3)
Level IV	659 (6.0)	11 (0.1)	134 (1.2)	633 (5.7)	429 (3.9)	1,866 (16.9)
N.D.*	1,631 (14.8)	41 (0.4)	781 (7.1)	325 (2.9)	40 (0.4)	2,818 (25.5)
Total	5,770 (52.2)	331 (3.0)	1,790 (16.2)	2,690 (24.3)	472 (4.3)	11,053 (100)

PERCENTAGES IN PARENTHESES REFER TO PROPORTION OF THE WHOLE STUDY SAMPLE; THE CONCORDANT PAIRS REPRESENT PATIENTS WHO WERE NOT TRANSFERRED OR TRANSFERRED FROM ONE HOSPITAL OF A GIVEN LEVEL TO ANOTHER HOSPITAL OF THE SAME LEVEL;

* N.D. = NONDESIGNATED HOSPITALS THAT ARE NOT INCLUDED IN THE TRAUMA SYSTEM. BY DEFINITION, PATIENTS SEEN IN THESE HOSPITALS AND NOT TRANSFERRED TO A HOSPITAL INCLUDED IN THE TRAUMA SYSTEM ARE NOT INCLUDED

Except for PTC, the majority of fatalities occurred within the first 6 h of admission [Table 3], and deaths occurred within the first 2 h more frequently at level IV and level III centers (*P* < 0.001).

**Table 3:** Death delay of injured children by trauma center type

Time from arrival to death	PTC *n* (%)	Level I *n* (%)	Level II *n* (%)	Level III *n* (%)	Level IV *n* (%)	Total *n* (%)
<2 h	6 (6.0)	10 (43.5)	17 (41.5)	60 (72.3)	23 (82.1)	116 (42.1)
2–5 h	12 (12.0)	2 (8.7)	3 (7.3)	8 (9.6)	1 (3.6)	26 (9.5)
6–11 h	11 (11.0)	1 (4.3)	1 (2.4)	3 (3.6)	1 (3.6)	17 (6.3)
12–47 h	36 (36.0)	6 (26.1)	5 (12.2)	1 (1.2)	2 (7.1)	50 (17.5)
≥48 h	31 (31.0)	4 (17.4)	11 (26.8)	6 (7.2)	1 (3.6)	53 (19.4)
Missing	4 (4.0)	0 (0)	4 (9.8)	5 (6.0)	0 (0)	13 (5.1)
Total	100	23	41	83	28	275

Mortality was related to transfer status and, since transfer status was related to level of care [Table 1], the variable transfer was a potential confounder and added to the model as a dichotomous variable. The area under the ROC curve was 0.981, attesting the very high discrimination of this model.

Pediatric trauma mortality was associated with trauma center type [Table 4]. Compared with children treated at PTC, all other levels of care experienced higher mortality (*P* < 0.01).

**Table 4:** Adjusted odds ratios of pediatric trauma death by logistic regression

Variables	Adjusted* OR (95% CI)
Trauma center type	
Level IV	9.85 (2.35–41.32)
Level III	5.24 (2.10–13.08)
Level II	2.52 (1.27–5.00)
Level I	3.08 (1.26–7.52)
PTC (reference)	1
Age	
<1 year	2.61 (1.15–5.93)
1–4 years	0.99 (0.47–2.10)
5–9 years	0.83 (0.46–1.49)
10–16 years (reference)	1
GCS	0.65 (0.60–0.70)
SBP	0.34 (0.23–0.49)
RR	0.35 (0.24–0.51)
NISS	1.06 (1.05–1.07)
Transfer	0.98 (0.57–1.69)

OR, ODDS RATIO; CI, CONFIDENCE INTERVAL; SBP, SYSTOLIC BLOOD PRESSURE; RR, RESPIRATORY RATE; NISS, NEW INJURY SEVERITY SCORE;

* ADJUSTED SIMULTANEOUSLY FOR ALL VARIABLES IN THE TABLE

Restriction to nonearly deaths (i.e., admission-death ≥6 h), the highest severity (i.e., ISS >15) and head trauma did not influence the results: mortality remained lower at PTC [Table 5].

**Table 5:** Odds ratios (OR) of pediatric trauma death for three subgroups: Early deaths excluded, severe trauma patients and head-injured patients

Subgroups	Adjusted OR* (95% CI)				
	PTC	Level I	Level II	Level III	Level IV
Early deaths excluded (admission–death ≥6 h; n = 10,911)	1	3.0 (1.2–7.8)	2.6 (1.3–5.3)	6.5 (2.4–17.9)	9.8 (1.9–49.5)
Severe trauma patients (ISS >15; n = 1,700)	1	2.2 (0.8–6.1)	1.5 (0.7–3.5)	9.9 (2.3–43.3)	31.1 (3.6–268.8)
Head-injured patients (n = 3, 169)	1	2.3 (0.8–6.6)	1.9 (0.9–4.4)	6.7 (1.6–27.5)	26.0 (3.6–190.5)

* ADJUSTED FOR AGE, GCS, SYSTOLIC BLOOD PRESSURE, RESPIRATORY RATE, NEW INJURY SEVERITY SCORE AND TRANSFER

## DISCUSSION

The focus of the present study was to compare the outcome of injured children treated at PTC and ATC in an inclusive trauma system where long distances prohibit the immediate and direct transport of injured children directly to the PTC in most circumstances.

Our first objective was to determine where pediatric trauma patients are treated in such a system. Our results show that, indeed, pediatric trauma patients are most often initially received in hospitals that are not PTC but that the most severely injured are indeed transferred to PTC. Our data support the idea that the Quebec inclusive trauma system works in two ways: first, by revealing that ATC in the major metropolitan area of Montreal (where both PTC and ATC are available) receive almost no pediatric trauma patients and secondly, by the fact that despite very long distances involved elsewhere, more than half of all injured children in the QTR were treated at PTC. It is also expressed by the volume of transfers, the fact that children with higher ISS are more often treated in PTC.

Our second objective was to compare the mortality between the different types of trauma centers. This study suggests that in our system, injured children treated at PTC have significantly lower mortality than those treated at each of the four levels of ATC. These results emphasize the need to define the appropriate role for each level of trauma center within an inclusive trauma system in the management of injured children.

Other authors have compared PTC and ATC mortality among children. Nakayama *et al*.[15] found no difference in standardized mortality ratios generated by TRISS methodology between injured children treated at PTC and urban ATC in Pennsylvania. The authors hypothesized that similarity of survival at urban ATC and PTC was probably due to the triage system applied in an urban environment: young children and those with head injuries were routinely triaged to PTC, and these were the two factors that affected the survival of the injured children.

Using the Pennsylvania Trauma Outcome Study registry, Potoka *et al*.[16] concluded that children treated at PTC or ATC with added qualifications in pediatrics had better survival than those treated at level I or level II ATC, a finding similar to ours. However, their study was criticized for lack of adjusting for differences in injury severity, asserting that median of ISSs (RTS and GCS) were similar across trauma center types.

On the other hand, Sherman *et al*.[17] examined data from the Pennsylvania Trauma Outcome Study registry using a variation on TRISS-predicted outcome (the difference between actual survival and predicted survival), and found improved survival for children treated at level I ATC and ATC with added qualifications in pediatrics when compared with those treated at PTC and level II ATC. Although Sherman *et al*. applied rigorous statistical methods, their analysis could be compromised by use of TRISS methodology, which is criticized by authors for the low-predictive accuracy that could lead to suboptimal risk adjustment.[31–33]

Like in this study, Osler *et al*.[18] used a logistic regression model to compare the mortality of injured children treated at PTC and ATC. Variables included in their model were those associated with the survival of injured children and available in the National Pediatric Trauma Registry (age, ISS, Pediatric Trauma Score,[34] trauma mechanism). No significant difference in survival between children treated at ATC versus PTC was found. These results could be attributable to the fact that the National Pediatric Trauma Registry operates on a voluntary basis. Therefore, contributing trauma centers are likely to have a significant interest in pediatric trauma care. The fact that no difference in survival was observed may also be due to inclusion in the ATC group of centers with additional qualifications in pediatrics and whose pediatric trauma care quality is comparable with PTC. Our study adds to the former by including sensitivity analysis for the subgroups of more severely injured children (*n* = 1,700) and head-injured children (*n* = 3,169). Better survival at PTC was also observed among these subsamples of children. A recent study using the Florida trauma system data also demonstrated that severely injured children treated at PTC had a survival advantage compared with those treated at nonpediatric trauma centers.[35]

### Limitations and strengths

The results of this study could have been affected by information bias. QTR data are subject to rigorous data collection and validation procedures. We therefore believe that its data quality is good. However, missing information on the initial GCS and RR is a concern. The GCS is often missing from trauma registries, and the problem is not exclusive to this study. In published North American trauma registry reports and trauma studies, proportions of observations with missing GCS vary from 9% to 38%.[36–39]. In this study, 53.6% of our data observations had no recorded initial GCS, which is higher than that usually reported in other studies. The missing data situation was handled with multiple imputation, which has been shown to generate unbiased effect estimates.[40] This was confirmed by simulations of Moore *et al*.[41] that showed that using patients with imputed values provided less-biased results than exclusion of patients with missing values. Moreover, multiple imputation has been found to be adequate for use, specifically in studies of pediatric trauma patients.[42,43] In addition, results were similar when patients with missing GCS or RR were excluded from analyses.

Information about time from injury to prehospital provider arrival on scene or initial transport from scene to receiving hospital is not included in the QTR. As transport time is related to center type, and has also been reported to be associated with survival,[44,45] this variable is a potential confounder. We could not adjust for transport time in our model. This bias could lead to penalisation of hospitals in rural areas as they may receive patients a long time after the occurrence of injury. It would be unlikely, however, to entirely explain the strong association observed. It is also possible that variables used for case mix control are not appropriate for the pediatric population. For example, we used the RTS (which has been validated in the pediatric trauma population as a significant predictor of in-hospital mortality[46–48]) instead of the Pediatric Trauma Score, which could not be used since it is not noted by ATC in our setting. However, it is unlikely that replacing the RTS with the Pediatric Trauma Score could have reversed the strong effect found, especially as with the exception of adult level I centers, patients treated at PTC were more severely injured than those treated elsewhere.

As the probability of death decreases over time, the better outcome at PTC is possibly biased by the fact that PTC admitted most of the transferred patients later in their course (73% of the patients treated at PTC were transfers and nearly half of them were transferred more than 12 h after admission at the referring hospitals). Nevertheless, after exclusion of early death, the mortality remained lower at PTC, and similar findings were observed in our outcome model adjusting for transfer delay. Similarly, the attribution of all outcomes to the receiving hospital in the case of transfers may introduce some bias as initial stabilization and treatment resulting in life saving may have been done at the primary center. However, a sensitivity analysis, whereby transfers were attributed to the first receiving hospital except if the transfer occurred in the first 2 h, still demonstrated better survival at PTC (data not shown). As combining the two PTC and the level I ATC with added pediatric qualifications could be questionable, analyses based on the original classification of the centers were performed and no difference in survival between these two types of centers was found (data not shown). Finally, the relatively low mortality among injured children implies that morbidity or functional outcome might be more sensitive markers of the quality of pediatric trauma care. This should be the subject of further research.

The strongest asset of this study is that it represents, to our knowledge, the first analysis of pediatric mortality in an inclusive trauma system. The study is also original as it deals with the specific situation of remote rural communities where direct transport to a PTC by the prehospital system is impeded by long distances.

## CONCLUSIONS

These results show that an inclusive trauma system can triage most severe pediatric trauma injuries to PTC, even when long distances are involved and that injured children treated at PTC have better survival than those treated at all other levels of ATC. The results of this study also suggest that severely injured children could benefit from transfer to PTC.

## Appendix

1. Characteristics of designated trauma centers in Quebec
  Level I trauma centers provide comprehensive multidisciplinary and definitive care to the severely injured adult or pediatric trauma patient. They have 24-h coverage by an emergency physician, general surgeon, neurosurgeon, anesthesiologist and orthopedist. All other surgical specialists are available within 30 min. Level I trauma centers have academic responsibilities for trauma-related education, training and research. Level II trauma centers serve as regional trauma centers, providing comprehensive, specialized and definitive trauma care for severely injured patients, including neurosurgery. Level III trauma centers provide initial evaluation and stabilization of injured patients and transfer patients requiring specialized care to higher-level trauma centers. Comprehensive trauma care is provided to those patients who can be maintained in a stable or improving condition. Level III centers are covered by emergency room staff on-site with a general surgeon and other specialists (anesthesiologists and orthopedists) on-call. By definition, all level III centers have intensive care units. Level IV centers have emergency room physician coverage, with a general surgeon on-call and promptly available for major resuscitations; they provide initial evaluation, resuscitation and stabilization, including emergency surgery of critically injured patients prior to transfer.
2. Multiple imputation of missing physiological data
  Like most trauma registries,[36,39] the QTR suffers from a proportion of missing physiological data. GCS was missing for 53.6% of the data observations. According to emergency physicians, trauma surgeons and archivists working on the registry, the most common cause of missing scores was the fact that the GCS was not recorded for patients with evident minor extracranial trauma and normal level of consciousness. Prehospital endotracheal intubation and/or sedation are not performed in the province of Quebec. SBP was missing for 18% of data observations and RR was not recorded for 35% of patients. Data were not missing for other predictors used in the survival model.
  Compared with those with missing physiological data, patients with complete data (*n* = 3,766; 34.1% of the cohort study) had higher injury severity (28.7% versus 8.5% with ISS >15), were more likely to have a head injury (respectively, 51.6% versus 16.8%) and also had higher mortality (3.5% versus 2.0%).
  A solution to the problem of missing data is to predict missing values from other information available in a patient file. This is known as data imputation and can be performed with several methods, the most interesting of which is multiple imputation. This method and the results of a simulation study demonstrating its validity in a study population of the QTR are explained in detail in a previous publication.[41] We hypothesized (as it can never formally be verified) that data in the QTR are probably missing at random manner as other registry variables will provide some but not all information on missing data. Missing physiological values were thus imputed with multiple imputation. Briefly, an imputation model was constructed using all trauma registry variables related to either the GCS/SBP/RR or the fact that they were missing. The variables used in the imputation process were age (with dummy variables), injury mechanism (with dummy variables), NISS (continuous), maximum head AIS (0–6), GCS (semicontinuous), SBP (continuous), RR (log transformed), transfer (dichotomous), disposition at discharge from the ED (with dummy variables), level of care (with dummy variables) and discharge destination (with dummy variables).
  The Markov Chain Monte Carlo method with a single chain was used to impute five possible values for each missing GCS/SBP/RR and the results were combined using the method described by Little and Rubin.[40] Imputation was performed with PROC MI in SAS^®^. Analyses were performed on the five data sets generated by the imputation process and combined using PROC MIANALYZE.
3. Model used for the analysis
  $\mathrm{og}\frac{\pi_{ij}}{1-\pi_{ij}}$ = α_j_ + β_1_AGE_0-<1 ij_ + β_2_AGE_1-4 ij_ + β_3_AGE_5-10 ij_ + β_4_AGE_11-16 ij_ + β_5_NISS_ij_ + β_6_GCS_ij_ + β_7_RRrts_0 ij_ + β_8_RRrts_1-5 ij_ + β_9_RRrts_6-9 ij_ + β_10_RRrts_10-29 ij_ + β_11_RRrts_>29 ij_ + β_12_SBPrts_0 ij_ + β_13_SBPrts_1-49 ij_ + β_14_SBPrts_50-75 ij_ + β_15_SBPrts_76-89 ij_ + β_16_SBPrts_>89 ij_ + β_17_PTC_ ij_ + β_18_ATCI_ ij_ + β_19_ATCII_ ij_ + β_20_ATCIII_ ij_ + β_21_ATCIV_ ij_
